# Seroprevalence of *Toxoplasma gondii* and associated risk factors in domestic pigs raised from Cuba

**DOI:** 10.1007/s00436-021-07245-1

**Published:** 2021-07-27

**Authors:** Julio César Castillo-Cuenca, Álvaro Martínez-Moreno, José Manuel Diaz-Cao, Angel Entrena-García, Jorge Fraga, Pedro Casanova Arias, Sonia Almería, Ignacio García-Bocanegra

**Affiliations:** 1 Departamento, de Medicina Veterinaria Y Zootecnia, Facultad de Ciencias Agropecuarias, Universidad Central “Marta Abreu” de Las Villas, Villa Clara, 54830 Santa Clara, Cuba; 2 Animal Health and Zoonosis Research Group (GISAZ), Department of Animal Health, University of Cordoba, 14014 Córdoba, Spain; 3 Department of Animal Health (Parasitology and Parasitic Diseases), University of Cordoba, 14014 Córdoba, Spain; 4 Departamento de Parasitología, Centro Nacional Para La Producción de Animales de Laboratorio (CENPALAB), 10300 La Habana, Cuba; 5 Departamento de Parasitología, Instituto de Medicina Tropical Pedro Kourí, 11400 La Habana, Cuba; 6 Department of Health and Human Services, Food and Drug Administration, Center for Food Safety and Nutrition (CFSAN), Office of Applied Research and Safety Assessment (OARSA), Division of Virulence Assessment, Laurel, MD USA

**Keywords:** *Toxoplasma gondii*, Seroprevalence, Domestic pigs, Public health, Cuba

## Abstract

A cross-sectional study was carried out to determine the seroprevalence of *Toxoplasma gondii* and associated risk factors in pigs in the largest pork-producing region in Cuba. Serum samples from 420 pigs, including 210 sows and 210 post-weaning pigs, were tested for antibodies against *T. gondii* using a commercial indirect enzyme-linked immunosorbent assay. Anti-*T. gondii* antibodies were detected in 56 animals (13.3%, 95% CI: 10.1–16.6). A generalized estimating equations model revealed that the risk factors associated with higher seropositivity in pigs were altitude (higher in farm’s location < 250 m above sea level (masl) versus ≥ 250 masl) and age (higher in sows compared to post-weaning pigs). The results indicated that this protozoan parasite is widely distributed on pig farms in the study area, which is a public health concern since the consumption of raw or undercooked pork meat products containing tissue cysts is considered one of the main routes of *T. gondii* transmission worldwide. Control measures should be implemented to reduce the risk of exposure to *T. gondii* in pigs in Cuba.

## Introduction

Toxoplasmosis is a worldwide zoonotic disease caused by the obligate intracellular protozoan parasite, *Toxoplasma gondii*, which infects virtually all warm-blooded species including human beings (Dubey et al. 2020). Approximately one-third of the human population is considered to be infected by this protozoan parasite (Behnke et al. 2020). Although *T. gondii* infection is usually asymptomatic, it can cause abortion, as well as blindness, neuromuscular disease, and even death in immunocompromised people (Dubey 2010). Moreover, an association between toxoplasmosis and neuropsychiatric disorders, including schizophrenia, has been suggested (Flegr and Horáček 2017).

*Toxoplasma gondii* is an important food-borne pathogen (EFSA 2018). The consumption of raw or undercooked meat products containing tissue cysts is considered one of the main routes of *T. gondii* transmission worldwide (Dubey 2010; Almeria and Dubey 2021). In this respect, pork is one of the major sources of human toxoplasmosis in some countries (Dubey et al. 2020; Almeria and Dubey 2021). Public concerns associated with *T. gondii* clearly indicate the need for epidemiological investigation in animals that can be used as a source of food. Previous immunological studies on the presence of *T. gondii* antibodies in domestic pigs carried out in Latin America showed wide variations in the seroprevalence among countries and between regions within the same country (Cañón-Franco et al. 2014; Feitosa et al. 2014; Foroutan et al. 2019; Dubey et al. 2020). In Cuba, anti-*T. gondii* antibodies have been found in human patients with acquired immunodeficiency syndrome (Alfonso et al. 2009) and retinochoroiditis (Regalado Andújar et al. 2013), in pregnant women (González-Morales et al. 1995), neonates (Goya Batista et al. 2014), and blood donors (Sánchez-Artigas et al. 2009). Seropositivity has also been reported in domestic animals, including cats (Grandía et al. 2012) and dogs (Navarrete et al. 2017) in this country. Nevertheless, information regarding *T. gondii* in domestic pigs in Cuba is very scarce. The only previous survey in this species was carried out in Ciego de Ávila province (Central Cuba) between 1980 and 2002 (Suárez-Hernández et al. 2005). Hence, the aim of this study was to assess the current seroprevalence and risk factors associated with *T. gondii* in pigs in the largest pork-producing region in Cuba.

## Material and methods

### Study design

**Fig. 1:**
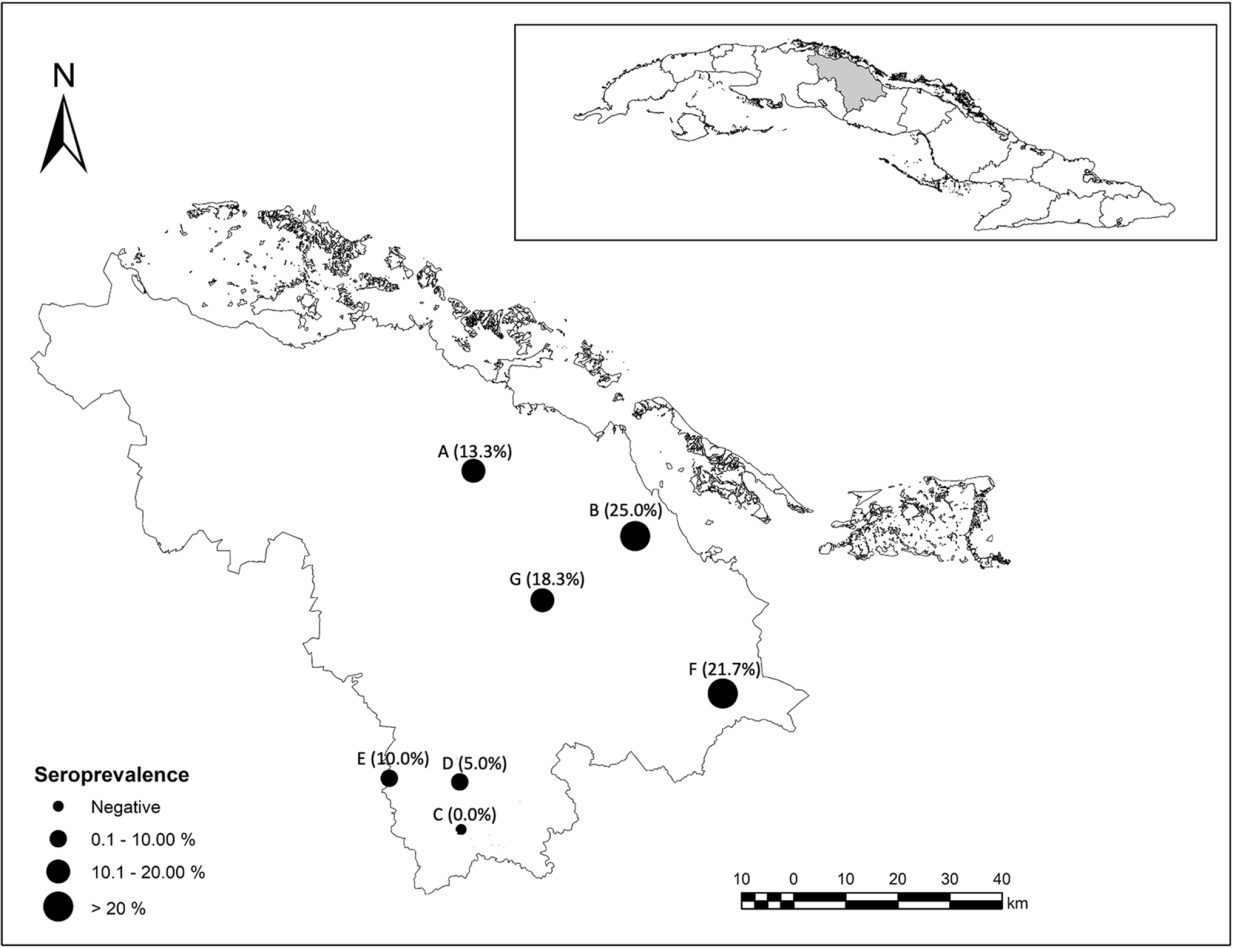
Map of Villa Clara province (Cuba) showing the distribution and within-farm seroprevalence of *Toxoplasma gondii* on the sampled pig farms

A cross-sectional study was carried out to determine seroprevalence against *T. gondii* in domestic pigs in Villa Clara (Central Cuba) (Fig. 1). This region accounts for the highest number of domestic pigs and is the largest pork producer in Cuba, with annual production at around 49,332 tons (ONEI 2017). Pork production in Cuba is characterized by an agreement management system. This means that all breeding farms, including sows, reproductive males, and piglets (from post-farrowing to post-weaning), are managed by the State government in intensive production systems. Fattening is then carried out by private swine farmers until the pigs are ready for slaughter.

The total population of all breeding farms in the study area was used to calculate sampling size. The size of the sampled farms ranged from 500 to 1,600 sows (mean: 1,050 sows). The breeds of the sows and post-weaning pigs were Yorkshire/Landrace and Yorkshire/Landrace X CC21 (Cuban paternal breed), respectively. Sample size was calculated assuming a prevalence of 50% (which provides the highest sample size in studies with unknown prevalence) with a 95% confidence level (95% CI) and desired precision of ± 5%, resulting in 384 domestic pigs to be sampled (Thrusfield et al. 2018). Sixty animals, including 30 sows and 30 post-weaning pigs, were randomly selected from each pig farm in order to detect infection with 95% probability and assuming a minimum within-farm prevalence of 6%. A total of 420 pigs were finally sampled in seven (A–G) farms managed under governmental intensive production management. All sampled farms presented very similar biosecurity measures including self-replacement gilts (replacement of breeding sows using gilts from the same herd), all-in-all out management, absence of cats, absence of other animal species, perimetral fence around the farm, rodent and insect control, disinfection and disinfestations protocols, sanitary ford, and water chlorination, among others. Farms A, B, F, and G were located < 250 m above sea level (masl), while farms C, D, and E were located at altitudes ≥ 250 masl.

### Sample collection and serological analysis

The collection of samples analyzed in the present study was part of the official Animal Health Campaigns under Cuban legislation. No animals were specifically sampled for this study; therefore, no ethical approval was necessary. Blood samples (about 10 ml) were collected using the orbital sinus puncture method. Samples were then centrifuged at 4,800 rpm for 10 min. Serum samples were separated and stored at − 20 °C until analysis. To obtain the presence of the antibodies against *T. gondii*, serum samples were analyzed using a commercial indirect ELISA (PrioCHECK® *Toxoplasma* Ab porcine, Thermo Fisher Scientific Prionics Lelystad BV) in accordance with the manufacturer’s recommendations (Castillo-Cuenca et al. 2020). The sensitivity and specificity of this ELISA according to the manufacturer are 98% and 99.6%, respectively.

### Statistical analysis

Individual seroprevalence against *T. gondii* was calculated as the ratio of seropositive animals to the total number of animals examined, using two‐sided exact binomial confidence intervals (95% CI). Analysis of means (ANOM) applied to proportions was used to identify farms with a significantly different within-farm seroprevalence relative to the overall mean combining all the sampled farms (“grand mean”), enabling detection of groups that deviate significantly from the overall mean (Rao 2005). The analysis was performed using the “ANOM” package (Pallmann and Hothorn 2016) of the statistical software R (R v. 3.5.2). If a statistically significant difference between the farms was found by ANOM, a *dummy* variable was created (“significantly different farm” vs “other farms”) and included in the bivariate analysis.

Epidemiological information including age, sex, farm (from A to G), altitude, and farm size was gathered for each sampled animal. For sows, data on offspring per birth, number of parities, weaning piglets, and stillbirths were also recorded. Bivariate chi-square and Fisher’s exact tests were performed to obtain the statistical significance of independent variables regarding individual *T. gondii* status (dependent variable). Variables with *P* < 0.20 in the bivariate analysis were selected as potential risk factors. Collinearity between pairs of variables was tested by Cramer’s *V* coefficient. Finally, a generalized estimating equation (GEE) was carried out to study the effect of the variables selected on the basis of bivariate analysis (Thrusfield et al. 2018). The number of seropositive animals was assumed to follow a binomial distribution and the “farm” was included as random effect. A logarithmic link function was considered. A forward introduction of variables was used, starting with the variable with the lowest *P*value in bivariate analysis. At each step, the confounding effect of the included variable was assessed by computing the change in the odds ratios (ORs). The model was re-run until all remaining variables presented statistically significant values (likelihood-ratio Wald’s test, *P* < 0.05) and a potential relationship with the response variable existed. The choice of the best model was based on the quasi-likelihood under independence model criterion (Hanley et al. 2003). Statistical analyses were performed using SPSS v25.0 software (Statistical Package for Social Sciences, Inc., Chicago, IL, USA).

## Results

**Fig. 2:**
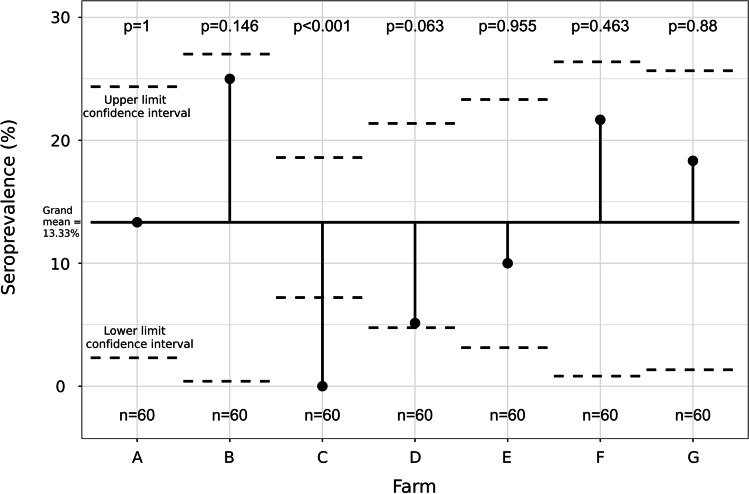
Results of the analysis of means comparing seroprevalences on the sampled farms

Antibodies against *T. gondii* were detected in 56 of 420 pigs tested (13.3%, 95% CI: 10.1–16.6). Seropositivity was found in six of the seven (85.7%) tested farms, and the within-farm seroprevalence ranged between 5.0 and 25%: with the highest seroprevalence observed in pigs from farm B and the lowest value in pigs from farm D had the lowest value. Interestingly, antibodies against *T. gondii* were not found in samples from farm C (Table 1) (Fig. 1). ANOM showed a significant lower seroprevalence on farm “C,” which was negative to the presence of anti-*T. gondii* antibodies, in relation to the overall mean of the other farms tested (Fig. 2).

**Table 1:** Distribution of the prevalence of antibodies against *Toxoplasma gondii*, using ELISA, on pig farms in Villa Clara province (Cuba) by category. Variables with *P*-value < 0.20 in the bivariate analysis were included in the multivariate analysis (generalized estimating equation) to determine potential risk factors

Variable	Categories	Number/overall (% positive)	OR	95% CI	Chi-square	*P*-value
Farm	A	8/60 (13.3)	NA		25.385	< 0.001
	B	15/60 (25.0)				
	C	0/60 (0.0)				
	D	3/60 (5.0)				
	E	6/60 (10.0)				
	F	13/60 (21.7)				
	G	11/60 (18.3)				
Altitude (m above sea level)	< 250	47/240 (19.6)	4.627	2.20–9.72	18.830	< 0.001
	≥ 250	9/180 (5.0)	*			
Farm size	> 501	23/120 (19.2)	1.918	1.07–3.43	4.497	0.022
	< 500	33/300 (11.0)	*			
Age	Sows	46/210 (21.9)	5.610	2.75–11.5	26.703	< 0.001
	Post-weaning pigs	10/210 (4.8)	*			
Sex	Male	4/106 (3.8)	0.627	0.17–2.29	0.505	0.350
	Female	6/102 (5.9)	*			
Offspring per birth	< 9	10/32 (31.3)	0.558	0.24–1.28	1.927	0.125
	≥ 10	36/178 (20.2)	*			
Parity number	< 3	30/116 (25.9)	0.588	0.30–1.16	1.927	0.125
	≥ 4	16/94 (17.0)	*			
Weaning piglets	< 9	10/47 (21.3)	1.05	0.48–2.31	0.014	0.541
	≥ 10	36/163 (22.1)	*			
Still birth	Yes	12/67 (17.9)	0.70	0.34–1.46	0.918	0.220
	No	34/143 (23.8)	*			

*NA* not applicable; *reference category

No association between seropositivity to *T. gondii* and sex, offspring per weaning piglets, and still birth was found in the bivariate analysis. Farm, altitude, farm size, age, offspring per birth, and parity number were selected for the multivariate analysis (Table 1). The final GEE model showed that the main factors associated with *T. gondii* seropositivity in pigs in Cuba were altitude and age. The prevalence of *T. gondii* antibodies was significantly higher on farm located < 250 masl (19.6%; 95% CI: 14.6–24.6) compared to the farms located at altitude ≥ 250 masl (5.0%; 95% CI: 1.8–8.2) (OR = 5.28; *P* = 0.001; 95% CI: 1.91–14.57). Significantly higher seropositivity was also found in sows (21.9%; 95% CI: 16.3–27.5) compared to post-weaning pigs (4.8%; 95% CI: 1.8–7.6) (OR = 6.05; *P* < 0.001; 95% CI: 2.53–14.60).

## Discussion

Consumption of contaminated undercooked or raw meat from farm animals has been known to be a major risk factor for acquisition of *T. gondii* infection in humans, and among food livestock species, pork is considered one of the main sources of *T. gondii* infection (Almeria and Dubey 2021). The first key step to prevent transmission of this zoonotic parasite in the swine production is to determine the presence of the parasite in the farms. In this regard, serological surveillance is the most commonly used method tool for identifying *T. gondii* exposure in pigs.

The individual seroprevalence detected in pigs raised in Cuba in our study (13%) is of the same magnitude as found previously in Cuba (14%) and in other Latin American countries such as Brazil (13%), Colombia (15%), and Mexico (ranging between 13 and 17%) (Suárez-Hernández et al. 2005; Foroutan et al. 2019; Dubey et al. 2020). Slightly higher mean seroprevalence values were observed in Brazil (ranging between 20 and 26%), while higher seropositivity was found in Argentina (48%), Brazil (ranging between 33 and 52%), Costa Rica (44%), Hawaii (49%), Mexico (ranging between 45 and 97%), Panamá (32%), and Peru (30%) (Cañón-Franco et al. 2014; Foroutan et al. 2019; Dubey et al. 2020). In contrast, lower seroprevalence rates were detected in other studies in Brazil (ranging between 0 and 8%), Chile (9%), and Mexico (ranging between 1 and 9%) (Foroutan et al. 2019; Dubey et al. 2020). Even though statistically accurate comparisons cannot be made given the differences in number of animals tested, the population sampled, and/or the different serological methods used, we would like to state that the seroprevalence in pigs in the study area should be considered moderate.

At least one seropositive pig was detected in six of the seven (85.7%) farms tested, with positive within-farm seroprevalence values ranging between 5.0 and 25.0%. Although farm “C” was negative to the presence of anti-*T. gondii* antibodies, the number of samples collected in each farm was calculated assuming a minimum within-farm prevalence of 6% and therefore, the possibility of that particular farm having a seroprevalence lower than 5% could not be discounted. The results indicated that *T. gondii* infection is widespread on pig breeding farms in Cuba. Since the sampled farms were all managed under a very similar production system, the environmental characteristics may explain differences in the seroprevalences in pigs within the study region. In this regard, farms located < 250 masl showed significantly higher seropositivity compared to the raised at higher altitude. Our results are in agreement with those reported by Villari et al. (2009) who consider the higher altitude (> 200 masl) of the farms as a protective factor of *T. gondii* exposure; this observation is likely associated with a reduced environmental viability of oocysts with decreasing ambient temperature and, perhaps, also humidity. Higher seroprevalence levels were also found in wild boars (*Sus scrofa*) sampled in hunting states located < 600 masl compared to those sampled at higher altitude (Calero-Bernal et al. 2016). In contrast, other studies observed higher seropositivity to *T. gondii* in pigs raised in mountainous regions than those raised in lowlands (Alvarado-Esquivel et al. 2012; Papatsiros et al. 2016). The reason for these differences is unclear; however, environmental, and climatic conditions may impact survival of oocysts in soil, food, and water contaminated with feline feces (Gauss et al. 2006), which are the likely sources of infection for pigs. Further studies are needed to address this issue.

Significantly higher seropositivity was found in sows compared to post-weaning pigs. Age is an important factor affecting *T. gondii* seroprevalence in pigs because most animals acquire *T. gondii* infection postnatally (Dubey 2009). The higher prevalence of *T. gondii* antibodies in sows compared to post-weaning pigs is consistent with those previously reported (García-Bocanegra et al. 2010a; Hill et al. 2014; Djokic et al. 2016; Castillo-Cuenca et al. 2020) and probably reflects the cumulative likelihood of exposure to *T. gondii* and lifelong persistence of IgG antibodies. Maternal-derived antibodies decline after the first week of age, but the decay is dependent on the antibody level of the dam at birth. However, because maternally transferred antibodies can persist until 4 months of age (Dubey 2009; García-Bocanegra et al. 2010b), the presence of maternally transferred antibodies detected in some seropositive post-weaning pigs cannot be ruled out.

Toxoplasmosis outbreaks have been reported in humans by ingestion of infected porcine meat containing tissue cysts (Choi et al. 1997; Vitale et al. 2014; Almeria and Dubey 2021). Even though we are not aware of any report of human toxoplasmosis directly linked to eating infected pork in Cuba, ocular toxoplasmosis (Mesa Hernández et al. 2011; Galbán Lueje et al. 2013; Bustillo et al. 2015; Ginorio Gavito et al. 2017), toxoplasmic encephalitis in patients with acquired immunodeficiency syndrome (Alfonso et al. 2009), acute toxoplasmosis in pregnant women (Lombana et al. 2012), and congenital toxoplasmosis (Amador Morán et al. 2016) have been reported in this country. Moreover, seropositivity values found in pregnant women (38%) (González-Morales et al. 1995), neonates (23%) (Goya Batista et al. 2014), and blood donors (13%) (Sánchez-Artigas et al. 2009) indicate that *T. gondii* is widely distributed in the human population in Cuba. Since viable *T. gondii* can be found in seropositive pigs, our results suggest that the consumption of non-properly cooked pork products may contribute to human toxoplasmosis in Cuba, and studies on the zoonotic impact of this disease are urgently needed in this country.

In summary, this is the first report on *T. gondii* in pigs in Villa Clara province, the largest pork-producing region in Cuba. Although the number of sampled farms was limited, the results obtained provide a first approximation to *T. gondii* exposure in domestic pigs in a country where there was no recent information in this animal species. The observed seropositivity indicates that this zoonotic parasite is widespread in pig breeding farms in the largest pork-producing region in Cuba. This finding indicates a public health concern because seropositive pigs can harbor tissue cysts in their meat, therefore representing a tentative zoonotic risk for consumers of raw or undercooked porcine meat or its products. In addition, evisceration and management of carcasses of infected pigs could also constitute a risk of infection for humans. Our results may contribute to the development of improved control strategies against *T. gondii* in this country. Further immunological and molecular studies on genotypes circulating in pig farms must be conducted to increase the knowledge of the epidemiology of *T. gondii* in Cuba.
